# LncRNAs at the Crossroads of Precision Nutrition and Cancer Chemoprevention

**DOI:** 10.3390/cancers18030430

**Published:** 2026-01-29

**Authors:** Camelia Munteanu, Revathy Nadhan, Sabina Turti, Eftimia Prifti, Larisa Achim, Sneha Basu, Alessandra Ferraresi, Ji Hee Ha, Ciro Isidoro, Danny N. Dhanasekaran

**Affiliations:** 1 Biology Section, Faculty of Agriculture, University of Agricultural Sciences and Veterinary Medicine Cluj-Napoca, 3-5 Manastur Street, 400372 Cluj-Napoca, Romania; camelia.munteanu@usamvcluj.ro (C.M.); alexandra.sabina.turti@student.usamvcluj.ro (S.T.); eftimia.prifti@student.usamvcluj.ro (E.P.); larisa-daniela.achim@student.usamvcluj.ro (L.A.); 2 Stephenson Cancer Center, University of Oklahoma Health Sciences Center, Oklahoma City, OK 73104, USA; revathy-nadhan@ou.edu (R.N.); sneha-basu@ou.edu (S.B.); jihee-ha@ou.edu (J.H.H.); 3 Department of Pathology, University of Oklahoma Health Sciences Center, Oklahoma City, OK 73104, USA; 4 Department of Health Sciences, Università del Piemonte Orientale, 28100 Novara, Italy; alessandra.ferraresi@med.uniupo.it (A.F.); ciro.isidoro@med.uniupo.it (C.I.); 5 Department of Cell Biology, University of Oklahoma Health Sciences Center, Oklahoma City, OK 73104, USA

**Keywords:** long non-coding RNAs (lncRNAs), precision nutrition, cancer, phytochemicals, nutritional epigenomics, micronutrients, omega-3 fatty acids, metabolic rewiring, chemoprevention

## Abstract

Cancer remains a leading cause of morbidity and mortality worldwide, and effective strategies for cancer prevention are urgently needed to complement therapeutic advances. While dietary factors are known to influence cancer risk, the molecular mechanisms that mediate inter-individual responses to nutritional exposures remain poorly defined. Emerging evidence identifies long non-coding RNAs (lncRNAs) as pivotal regulators of gene expression, chromatin organization, metabolic homeostasis, immune signaling, and cellular stress responses, the core processes that drive cancer initiation and progression and are highly sensitive to nutritional status. In parallel, advances in precision nutrition have highlighted how variability in genetics, metabolism, microbiome composition, and epigenetic landscapes shape dietary influences on cancer susceptibility. This review integrates these rapidly evolving fields by positioning lncRNAs as molecular conduits that translate dietary exposures into transcriptional and epigenetic programs governing cancer development, progression, and therapeutic vulnerability. We provide mechanistic evidence demonstrating how dietary bioactive compounds and micronutrients, including polyphenols [such as curcumin, resveratrol, epigallocatechin gallate (EGCG)], flavonoids, alkaloids such as berberine, omega-3 (ω-3) fatty acids, folate, vitamin D, probiotic metabolites (such as butyrate and propionate), and trace elements (such as selenium and zinc), modulate oncogenic and tumor-suppressive lncRNAs. These nutrient–lncRNA interactions influence cancer-relevant pathways controlling proliferation, epithelial–mesenchymal transition (EMT), inflammation, oxidative stress, and metabolic rewiring. We further discuss emerging lncRNA signatures that reflect nutritional and metabolic states, their potential utility as biomarkers for individualized dietary interventions, and their integration into liquid biopsy platforms. Leveraging multi-omics datasets and systems biology, we outline AI-driven frameworks to map nutrient–lncRNA regulatory networks and identify targetable nodes for cancer chemoprevention. Finally, we address translational challenges, including compound bioavailability, inter-individual variability, and limited clinical validation, and propose future directions for incorporating lncRNA profiling into precision nutrition-guided cancer prevention trials. Together, these insights position lncRNAs at the nexus of diet and cancer biology and establish a foundation for mechanistically informed precision nutrition strategies in cancer chemoprevention.

## Simple Summary

Nutrition shapes many of the biological pathways involved in cancer, yet people often respond very differently to the same foods or dietary supplements. Long non-coding RNAs have emerged as important regulatory molecules that influence gene expression, cellular metabolism, inflammation, and stress response—processes central to cancer development. Growing evidence indicates that these RNAs are very sensitive to dietary bioactive compounds, positioning them as a critical molecular interface between nutrition and cancer biology. In this review, we explore how natural bioactive substances, vitamins, minerals, and fatty acids modulate long non-coding RNAs with either oncogenic or tumor-suppressive functions. We also discuss how systems biology approaches and artificial intelligence can be used to map nutrient–RNA interactions and uncover actionable targets for cancer prevention. By integrating nutritional science with molecular and computational frameworks, this review highlights the potential of long non-coding RNAs to inform more personalized and effective dietary strategies for reducing cancer risk.

## 1. Introduction

Cancer development and progression are driven by coordinated disruptions in gene regulation, chromatin architecture, cellular metabolism, immune signaling, and stress-adaptive responses [1,2,3,4]. While genetic alterations initiate malignant transformation, sustained tumor growth and progression are shaped by dynamic regulatory networks that integrate intrinsic molecular programs with environmental cues [1]. Among the regulatory layers that orchestrate these processes, long non-coding RNAs (lncRNAs) have emerged as central determinants of oncogenic and tumor-suppressive states. By functioning as molecular scaffolds, guides, decoys, and regulatory effectors, lncRNAs influence transcriptional control, epigenetic remodeling, RNA stability, and signaling pathway integration, virtually across all hallmarks of cancer [1,4,5,6].

Dysregulated lncRNA expression contributes to cancer initiation, epithelial–mesenchymal transition (EMT), metabolic rewiring, immune evasion, and therapeutic resistance [5,6,7]. Numerous lncRNAs act as context-dependent regulators that amplify oncogenic signaling pathways or reinforce tumor-suppressive checkpoints, thereby shaping cancer susceptibility and disease trajectory [8]. Importantly, lncRNAs exhibit cell type-specific, tissue-restricted, and environmentally responsive expression patterns, positioning them as sensitive molecular integrators of external stimuli [9,10]. These properties distinguish lncRNAs from protein-coding genes and underscore their relevance as mechanistic drivers, biomarkers, and potential therapeutic targets in cancer biology [11].

An emerging body of evidence indicates that lncRNA regulatory networks are highly responsive to nutritional and metabolic inputs [12]. Natural dietary compounds, including polyphenols, alkaloids, fatty acids, vitamins, probiotics, postbiotics, and trace elements, have been shown to modulate the expression and activity of oncogenic and tumor-suppressive lncRNAs across multiple cancer types [13,14]. Such nutrient-dependent modulation influences pathways central to carcinogenesis, including proliferation, inflammation, oxidative stress responses, EMT, stemness, and metabolic adaptation [15]. These findings suggest that diet-derived bioactive molecules do not act solely through classical antioxidant or anti-inflammatory mechanisms but can directly reprogram cancer-relevant transcriptional and epigenetic circuits via lncRNA-mediated regulation [16].

In parallel, advances in precision nutrition have highlighted the importance of inter-individual variability in shaping biological responses to dietary exposures [17]. Genetic background, metabolic phenotype, microbiome composition, and epigenetic state, collectively determine how nutritional signals are interpreted at the molecular level [18]. Within this framework, lncRNAs emerge as plausible mechanistic interfaces through which dietary factors influence cancer susceptibility and progression [19,20,21]. The convergence of cancer biology, lncRNA regulation, and precision nutrition highlights the potential of nutrient-responsive lncRNAs as molecular targets and biomarkers for cancer chemoprevention. “Chemoprevention” is used here in an expanded sense to include dietary bioactive compounds, in addition to pharmacologic agents, that modulate molecular pathways involved in cancer development. In this context, nutrients such as polyphenols, fatty acids, and vitamins influence gene regulation, epigenetic programs, inflammation, and cellular metabolism. Nutrition-based chemoprevention therefore refers to the strategic use of dietary components to reduce cancer risk or delay disease progression rather than to treat established malignancy.

Technological advances in multi-omics profiling, single-cell analysis, and systems biology are beginning to resolve the complexity of lncRNA-centered regulatory networks [22]. These approaches reveal how lncRNAs operate within interconnected molecular circuits that integrate environmental, metabolic, and inflammatory cues [19]. Moreover, computational modeling and artificial intelligence (AI) now provide powerful tools to map nutrient–lncRNA–pathway relationships, identify regulatory hubs, and predict context-specific responses that are difficult to capture through reductionist experimental strategies alone [23].

In this review, we synthesize current knowledge on the role of lncRNAs as molecular mediators linking nutrition to cancer biology. We focus on mechanistic insights into how dietary bioactive compounds and micronutrients modulate lncRNA-driven regulatory pathways underlying cancer genesis and cancer prevention. By integrating experimental evidence with systems-level and computational perspectives, we provide a conceptual foundation for lncRNA-guided precision nutrition strategies in cancer chemoprevention [17,21,24,25]. Understanding how modifiable dietary factors influence lncRNA regulatory circuits may ultimately lead to individualized approaches to reduce cancer risk and to establish prevention-focused translational strategies [26,27].

## 2. LncRNAs as Orchestrators of Cancer Initiation and Progression

**Figure 1:**
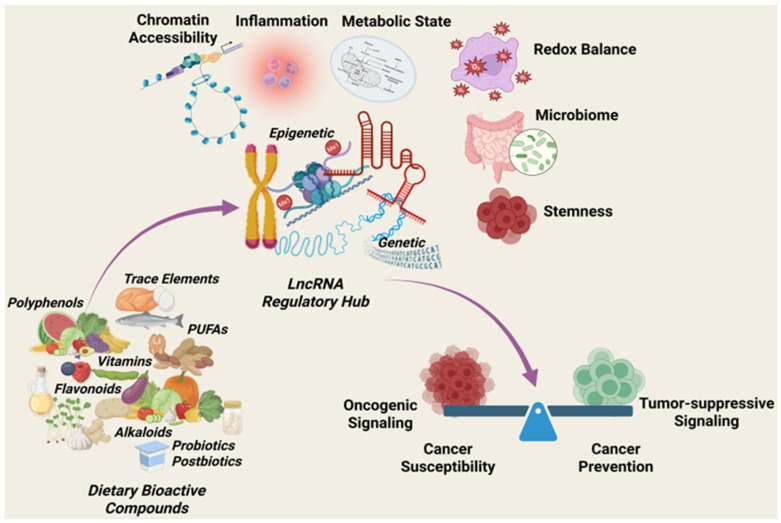
LncRNAs integrating nutrition with cancer susceptibility and prevention. Nutritional inputs, including polyphenols, flavonoids, alkaloids, polyunsaturated fatty acids (PUFAs), vitamins probiotics, postbiotics, and trace elements from the diet, modulate lncRNA expression and function within genetic and epigenetic contexts. As regulatory hubs, lncRNAs integrate these signals to shape key biological processes, including metabolic state, inflammation, chromatin accessibility, redox balance, microbiome interactions, and cellular stemness. These upstream effects converge on cell signaling pathways, shifting the balance between oncogenic and tumor-suppressive programs and ultimately influencing cancer susceptibility or prevention.

LncRNAs operate at the intersection of transcriptional control, epigenetic regulation, and signal integration, enabling them to coordinate complex cellular responses to both intrinsic and environmental cues [6]. In cancer, dysregulation of lncRNA expression and function contributes to all the hallmarks of malignancy, positioning these molecules as central regulators of oncogenic and tumor-suppressive states [7,28]. This section outlines the biological features of lncRNAs, their mechanisms of action, and their context-dependent roles in cancer-relevant regulatory networks, thus providing a mechanistic foundation for understanding how nutritional inputs can reshape lncRNA-driven programs. An overview of how dietary bioactive compounds interface with lncRNA-mediated regulatory networks to influence cancer susceptibility and prevention is illustrated in Figure 1.

### 2.1. Biogenesis, Classification, and Regulatory Modalities of LncRNAs

LncRNAs are RNA transcripts longer than 200 nucleotides that lack protein-coding capacity yet exert diverse regulatory functions across the genome [12]. They arise from heterogeneous genomic contexts, including intergenic regions, introns, antisense strands, and overlapping loci with protein-coding genes [29,30]. Although many lncRNAs share canonical features with messenger RNAs (mRNAs), such as RNA polymerase II-mediated transcription, splicing, and polyadenylation, their processing efficiency, subcellular localization, and stability are highly variable, contributing to context-dependent regulatory activity [6,31]. This intrinsic plasticity allows lncRNAs to serve as dynamic regulators of gene expression programs [12,32].

Functionally, lncRNAs can be classified based on their molecular modes of action as signals, decoys, guides, and scaffolds [29]. Signaling lncRNAs reflect tightly regulated transcriptional states and carry temporal or spatial information in response to cellular cues. Decoy lncRNAs sequester transcription factors [33], RNA-binding proteins, or microRNAs (miRNAs), thereby buffering or redirecting signaling outputs [34]. Guide lncRNAs recruit chromatin-modifying complexes to specific genomic loci, directing epigenetic remodeling and transcriptional control [35]. Scaffold lncRNAs assemble multi-protein complexes, facilitating coordinated regulation of chromatin structure, transcription, or post-transcriptional processes [36,37]. In addition, many lncRNAs have been shown to act as competing endogenous RNAs (ceRNAs) by harboring miRNA response elements that enable binding of specific miRNAs. Through sequestration of these miRNAs, lncRNAs can reduce their effective availability, thereby attenuating miRNA-mediated repression of downstream gene expression. This post-transcriptional regulatory mechanism is highly context dependent and can contribute to the modulation of gene networks involved in cancer progression and other cellular processes [5,6,7,8,9]. These fundamental mechanisms often coexist within a single lncRNA, underscoring the modular and multifunctional nature of lncRNA-mediated regulation [12,29,37].

### 2.2. LncRNAs in Cancer Hallmarks

Aberrant lncRNA expression is now recognized as a pervasive feature of cancer, contributing to nearly all established hallmarks of malignancy [1,11]. Numerous oncogenic lncRNAs promote sustained proliferative signaling by reinforcing pathways such as PI3K–AKT, MAPK, Wnt/β-catenin, and STAT3, while tumor-suppressive lncRNAs restrain cell cycle progression and maintain genomic integrity [38,39]. Through interactions with transcriptional regulators, chromatin modifiers, and RNA-binding proteins, lncRNAs fine-tune the amplitude and duration of growth-promoting signals [38,40].

LncRNAs also play central roles in EMT, a critical process underlying invasion and metastasis [41]. By regulating EMT-associated transcription factors, cytoskeletal remodeling, and cell adhesion programs, lncRNAs facilitate phenotypic plasticity that enables cancer cells to disseminate and colonize at distant sites [42]. In parallel, lncRNAs modulate inflammatory and stress-responsive pathways, shaping tumor-promoting microenvironments characterized by chronic cytokine signaling, oxidative stress, and immune suppression [43,44].

Metabolic reprogramming represents another major axis of lncRNA-mediated regulation in cancer. LncRNAs influence glycolysis, lipid metabolism, mitochondrial function, and redox balance, thereby supporting anabolic growth and adaptation to metabolic stress [45,46,47,48]. In immune contexts, lncRNAs regulate antigen presentation, immune checkpoint expression, and immune cell differentiation, contributing to tumor immune evasion [49,50]. Collectively, these functions position lncRNAs as master regulators that integrate signaling, metabolism, and microenvironmental interactions to sustain malignant phenotypes [51].

### 2.3. Oncogenic and Tumor-Suppressive LncRNAs: Context Dependency and Regulatory Balance

A defining feature of lncRNA biology in cancer is its pronounced context dependency. Some lncRNAs function predominantly as oncogenic drivers, promoting proliferation, invasion, and therapy resistance, whereas others act as tumor suppressors that constrain malignant progression [30,52]. This functional polarity is dictated by cellular context, tissue type, subcellular localization, and interaction partners rather than intrinsic sequence features alone.

Oncogenic lncRNAs often amplify nutrient- and growth factor-responsive signaling pathways, reinforcing transcriptional programs associated with proliferation, EMT, and metabolic adaptation [7,53]. In contrast, tumor-suppressive lncRNAs frequently stabilize genome integrity, repress oncogenic transcriptional networks, or enhance stress-induced checkpoints [54,55]. Importantly, the balance between oncogenic and tumor-suppressive lncRNA activity is highly sensitive to environmental and metabolic perturbations, making lncRNA networks particularly vulnerable to dysregulation during early tumorigenesis [56,57].

This dynamic equilibrium highlights why lncRNAs cannot be interpreted as universally beneficial or detrimental [58]. Instead, they function within finely tuned regulatory circuits whose outputs depend on cellular state and external inputs. Disruption of this balance contributes to malignant transformation and disease progression [59].

### 2.4. Early LncRNA Dysregulation in Carcinogenesis: Implications for Prevention

Accumulating evidence indicates that lncRNA dysregulation occurs early during carcinogenesis, often preceding overt histological transformation [60,61,62]. Changes in chromatin accessibility, metabolic stress, and inflammatory signaling can induce shifts in lncRNA expression that prime cells for malignant conversion [12,63]. These early alterations suggest that lncRNAs function not only as markers of established cancer but also as active participants in the initiation phase of tumor development [64].

Because lncRNAs respond rapidly to cellular stress and environmental cues, they represent attractive targets for early intervention. Transitional lncRNA expression patterns have been identified that mark critical inflection points in cancer evolution, such as the transition from localized growth to invasive or metastatic disease [11,41]. Targeting lncRNA-mediated regulatory circuits at these early stages may offer opportunities to intercept tumor progression before irreversible malignant programs are established.

Together, these observations establish lncRNAs as central orchestrators of cancer initiation and progression. Their regulatory versatility, context sensitivity, and early involvement in tumorigenesis provide the biological rationale for exploring upstream modulators, such as dietary and metabolic factors, that influence lncRNA networks. This framework sets the stage for examining how nutrition-derived bioactive compounds intersect with lncRNA-driven cancer pathways, as discussed in the following sections.

## 3. Nutritional Regulation of LncRNAs: A Precision Modulation Framework

Nutrition represents a uniquely actionable upstream regulator capable of influencing metabolic state, inflammatory tone, and chromatin accessibility, processes that converge on lncRNA-mediated regulatory networks [14,65]. Within this context, dietary bioactive compounds can be viewed as precision inputs that modulate lncRNA-driven transcriptional and epigenetic programs relevant to cancer susceptibility and prevention [16,66].

### 3.1. Nutrition as a Modulator of LncRNA-Driven Oncogenic Pathways

Although genetic and epigenetic alterations initiate and sustain malignant transformation, cancer progression is profoundly influenced by modifiable environmental factors [65,67,68]. Among these, nutrition represents a uniquely actionable factor capable of shaping cellular metabolism, redox balance, inflammatory tone, and epigenetic regulation [69,70,71]. However, the molecular mechanisms by which dietary exposures influence cancer susceptibility and progression remain incompletely defined. Emerging evidence suggests that lncRNAs, owing to their context-dependent, multi-functional role, serve as key molecular intermediaries linking nutrition to cancer-relevant transcriptional programs [14,27,72,73].

Unlike protein-coding mRNAs, lncRNAs exhibit highly dynamic, tissue-specific, and stimulus-responsive expression patterns [12]. This plasticity allows lncRNA networks to adapt to environmental cues and reconfigure gene regulatory states. Nutrient availability, dietary bioactive compounds, and metabolite fluxes can influence chromatin accessibility, transcription factor activity, RNA stability, and subsequent activation of specific signaling pathways via distinct sets of lncRNAs [74,75,76]. These properties position lncRNAs as susceptible targets of nutritional modulation, thus identifying them as promising targets for precision-based cancer prevention strategies.

### 3.2. Precision Nutrition as a Context for LncRNA-Mediated Cancer Modulation

Precision nutrition extends beyond generalized dietary recommendations by recognizing that genetic variation, metabolic phenotype, microbiome composition, and epigenetic states collectively shape biological responses to dietary inputs [77,78,79]. Within this framework, uniform nutritional interventions are likely to generate heterogeneous outcomes, including variable effects on cancer risk. In this context, lncRNAs offer a mechanistic vantage point from where this variability can be understood, as their expression and function are tightly coupled to cellular context and environmental signals.

From a cancer biology perspective, precision nutrition is not simply a lifestyle paradigm but a strategy to selectively influence molecular networks that govern oncogenic and tumor-suppressive pathways [17]. Nutrient-responsive lncRNAs may act as molecular sensors that translate dietary exposures into epigenetic and transcriptional outputs relevant to cancer genesis and progression. Thus, incorporating lncRNA biology into precision nutrition frameworks provides a mechanistic basis for stratifying individuals according to molecular responsiveness rather than dietary intake alone.

### 3.3. Dietary Bioactive Compounds as Regulators of LncRNAs

A growing body of experimental evidence demonstrates that specific dietary bioactive compounds can modulate the expression and activity of lncRNAs implicated in cancer initiation, promotion, and progression. These compounds include polyphenols, flavonoids, alkaloids, polyunsaturated fatty acids (PUFAs), vitamins, probiotics, postbiotics, and essential trace elements [13,21,80,81]. Rather than acting through single linear pathways, these nutrients influence lncRNA-centered regulatory circuits that intersect with proliferation, EMT, inflammation, oxidative stress responses, metabolic rewiring, and stemness [5,65,82,83,84,85,86,87].

Importantly, dietary compounds have been shown to differentially regulate oncogenic versus tumor-suppressive lncRNAs, thereby shifting the balance of transcriptional programs toward growth restraint, stress resolution, or differentiation. In many cases, these effects are mediated through epigenetic mechanisms, including chromatin remodeling, DNA methylation, histone modification, and modulation of transcription factor accessibility [65]. Such findings support the concept that nutrition-derived molecules can act as non-toxic epigenetic modulators that can reshape and restrain cancer-related lncRNA networks.

### 3.4. Moving Beyond Antioxidant Paradigms Toward LncRNA-Centric Mechanisms

Traditional explanations for the cancer-preventive effects of dietary compounds focus primarily on antioxidant activity or generalized anti-inflammatory properties. While these mechanisms remain relevant, they do not fully explain the specificity and temporal kinetics of transcriptional changes observed in response to dietary interventions. Increasing evidence indicates that lncRNA-mediated regulation represents a higher-order mechanism through which dietary compounds exert context-dependent, spatio-temporal effects on the pathobiology of cancer [13,86,88,89].

By targeting lncRNA-driven regulatory hubs, dietary bioactive compounds can simultaneously influence multiple signaling pathways and cellular phenotypes. This network-level modulation differs from single-target pharmacological approaches and aligns with the complex, multifactorial nature of cancer development [81,90]. A lncRNA-centric perspective, therefore, redefines nutrition as a programmable epigenetic input capable of altering cancer trajectories through coordinated regulation of multiple gene expression networks.

## 4. Nutritional Modulation of LncRNAs: Molecular and Pathway-Level Insights

Experimental studies across diverse cancer models have shown that nutritional bioactive molecules influence lncRNA-centered regulatory circuits that control transcriptional programs, epigenetic remodeling, metabolic adaptation, and inflammatory signaling [14]. Rather than acting through isolated molecular targets, these compounds modify interconnected signaling networks by shifting the balance between oncogenic and tumor-suppressive lncRNAs [16,81,91], leading to changes in cancer hallmarks, including cell proliferation, EMT, stress responses, and immune modulation [83,91].

### 4.1. Polyphenols and Flavonoids as LncRNA-Modulating Agents in Cancer

**Figure 2:**
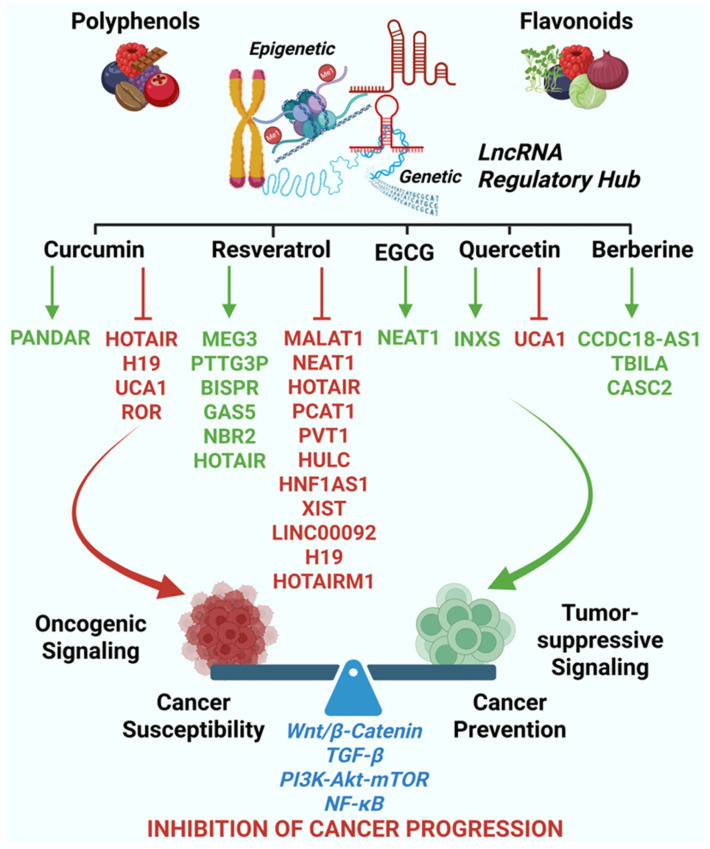
Modulation of lncRNAs by polyphenols and flavonoids. Polyphenols and flavonoids, including curcumin, resveratrol, epigallocatechin gallate (EGCG), quercetin, and berberine, selectively suppress oncogenic lncRNAs (e.g., HOTAIR, H19, MALAT1, NEAT1, UCA1, ROR) while inducing tumor-suppressive lncRNAs (e.g., GAS5, MEG3, NBR2, CASC2, INXS), converging on key regulatory pathways involved in proliferation, apoptosis, epithelial–mesenchymal transition (EMT), stemness, inflammation, and metabolic adaptation such as Wnt/β-catenin, TGF-β, PI3K–AKT–mTOR, and NF-κB signaling pathways. Red text denotes oncogenic lncRNAs, while green text denotes tumor-suppressive lncRNAs. Polyphenol-induced regulation of lncRNAs is concentration-, timing-, and context-dependent; therefore, individual lncRNAs are depicted according to their predominant reported functional role for clarity.

Polyphenols and flavonoids are among the most thoroughly studied groups of dietary bioactive compounds with anticancer effects. Beyond their well-known antioxidant and anti-inflammatory roles, growing evidence shows that these compounds also directly influence lncRNA expression and function (Figure 2), ultimately modifying transcriptional and epigenetic pathways that are key to carcinogenesis [13,81,92,93]. Many of these effects target lncRNAs that control proliferation, EMT, inflammation, oxidative stress responses, metabolic changes, and stemness [13,20,81,89,90,91,92,93,94]. Mechanistically, polyphenols modulate lncRNA networks through multiple pathways, including chromatin remodeling, DNA methylation, transcription factor accessibility, RNA stability, and disruption of lncRNA-protein complexes. Additionally, it has been shown that polyphenols such as resveratrol, pterostilbene, and genistein, may elicit anticancer activity through the non-coding RNA-mediated stimulation of protective autophagy in lung cancer [92]. Importantly, these regulatory effects often manifest as coordinated network-level reprogramming rather than isolated pathway inhibition, reinforcing the concept that lncRNAs act as integrative hubs for diet-responsive cancer signaling [93,94].

#### 4.1.1. Curcumin: Repression of Oncogenic LncRNAs and EMT-Associated Programs

Curcumin is one of the best-characterized polyphenols regarding lncRNA modulation in cancer. Several studies in renal cell carcinoma have shown that curcumin suppresses the oncogenic lncRNA HOTAIR, a key epigenetic regulator involved in chromatin remodeling, EMT, metastasis, and poor prognosis across various tumor types [94,95]. Downregulation of HOTAIR by curcumin is associated with decreased cancer cell migration and invasion, reduced expression of EMT markers and the restoration of epithelial traits in glioblastoma [96]. Curcumin also modulates the lncRNA PANDAR, which functions as a regulator of p53-dependent cell fate decisions [97]. In colorectal cancer, curcumin-induced upregulation of PANDAR promotes senescence rather than apoptosis, an effect that can be reversed upon PANDAR silencing, indicating a context-dependent role in growth arrest and stress adaptation [98]. In addition, curcumin suppresses other oncogenic lncRNAs, including H19, UCA1, and ROR, thereby disrupting Wnt/β-catenin–driven transcriptional programs linked to proliferation and stemness [13]. In gastric cancer cells, curcumin-mediated suppression of H19 reduces cellular proliferation through downregulation of c-Myc and concomitant upregulation of p53 [99]. In A549 lung cancer cells, curcumin significantly inhibits UCA1 expression, leading to attenuation of Wnt and mTOR signaling pathways, reduced cell proliferation, and enhanced apoptosis; importantly, these effects are reversed by UCA1 overexpression, confirming its central role in mediating curcumin’s anticancer effects [100]. Curcumin also suppresses ROR expression in prostate cancer stem cells, disrupting its miRNA sponge function toward miR-145 and preventing activation of Oct4, thereby reducing stemness, invasion, and tumorigenicity [101]. Consistent with these findings, curcumin inhibits Wnt/β-catenin signaling and tumor growth in hepatocellular carcinoma through downregulation of lncRNA ROR [102].

At the pathway level, curcumin-mediated lncRNA modulation intersects with TGF-β-dependent and independent EMT signaling, PI3K-AKT-mTOR activity, and NF-κB–driven inflammatory cascades. Through these coordinated effects, curcumin reprograms lncRNA-centered regulatory circuits that collectively restrain tumor progression [103,104,105].

#### 4.1.2. Resveratrol: Modulation LncRNA-Driven Proliferative and Stress–Response Pathways

Resveratrol exerts anticancer activity in part through repression of the oncogenic lncRNA MALAT1, a regulator of alternative splicing, transcriptional elongation, and metastatic competence [106]. Suppression of MALAT1 by resveratrol has been documented in gastric cancer, leading to reduced proliferation, impaired migration, and altered cell cycle progression [107].

In addition to MALAT1, resveratrol modulates NEAT1, a nuclear retained lncRNA involved in paraspeckle formation, stress adaptation, and therapy resistance. Downregulation of NEAT1 by resveratrol has been linked to disruption of the unfolded protein response signaling and sensitization to cellular stress, particularly in multiple myeloma and solid tumor models [80,108,109]. These effects position resveratrol as a modulator of lncRNA-mediated stress resilience rather than a direct cytotoxic agent.

In HT-29 colorectal cancer cells, resveratrol decreased the expression of lncRNAs H19, HOTAIR, PCAT1 and PVT1, that are associated with aggressive colon carcinomas, while it increased the expression of MALAT1 [110]. It is to be stressed that resveratrol modulates the expression of different lncRNAs depending on its concentration, the time of treatment, and the cell type. For instance, in SGC7901 gastric cancer cells, a 24 h treatment with 200 μM resveratrol increased the expression of MEG3, PTTG3P and BISPR, while that of GAS5 was significantly decreased. Interestingly, while 50 μM resveratrol increased the expression of H19 and MALAT1, a concentration of 200 μM resveratrol downregulated H19 expression [111]. Intriguingly, silencing the expression of H19 in these cells enhanced the antitumor effect of resveratrol [111].

Transcriptomic analysis of lncRNAs in ovarian cancer cell line OVCAR3, exposed to a clinically relevant dose of resveratrol (100 µM) for 24 h revealed significant modulation of numerous lncRNAs. Notably, there was a downregulation of oncogenic lncRNAs and an upregulation of tumor-suppressive lncRNAs [112]. Among the most upregulated lncRNAs were GAS5, NBR2, and HOTAIR, which are recognized for counteracting malignancy by promoting apoptosis, inhibiting cell proliferation, migration, and inducing autophagy [113,114,115,116]. In contrast, the most downregulated lncRNAs included H19, HULC, HOTAIRM1, and HNF1AS1, all of which are highly expressed in ovarian cancer and contribute to cell proliferation, migration, metastasis, glycolysis, and multidrug resistance [112]. Additionally, Resveratrol downregulated the expression of several other oncogenic lncRNAs such as XIST, LINC00092, and MALAT1 [112].

Collectively, resveratrol influences lncRNA-regulated pathways controlling oxidative stress, cell cycle checkpoints, cell metabolism, and epigenetic stability, reinforcing its role as a transcriptional and epigenetic regulator in cancer biology [117,118].

#### 4.1.3. EGCG, Quercetin, and Berberine: LncRNA Regulation Across Diverse Cancer Contexts

Epigallocatechin gallate (EGCG), the primary catechin in green tea, influences multiple lncRNAs involved in apoptosis, drug response, and inflammatory signaling. EGCG has been shown to upregulate NEAT1, facilitating increased cisplatin uptake by modulating CTR1 drug transporter expression, thus enhancing chemotherapeutic sensitivity in non-small cell lung cancer models [119]. EGCG also influences lncRNA-linked DNA methylation patterns, contributing to sustained transcriptional reprogramming [86].

Quercetin exerts antiproliferative effects through lncRNA-dependent mechanisms involving UCA1 and INXS. In breast cancer models, quercetin-mediated downregulation of UCA1 and induction of the pro-apoptotic lncRNA INXS promote G2/M cell cycle arrest and apoptosis [120]. These effects highlight quercetin’s ability to shift the balance between survival and death pathways through the targeted modulation of lncRNAs.

Berberine suppresses lncRNAs that promote EMT, invasion, and metabolic plasticity. Its anticancer effects are associated with the repression of mesenchymal transcriptional programs and reduced expression of EMT-associated genes, including Snail, BMP7, and NODAL, in part through lncRNA-mediated regulatory circuits, such as those involving lncRNA CASC2 [121]. Berberine also triggers mitochondrial stress and apoptotic signaling in malignant pleural mesothelioma cells, reinforcing its multifaceted impact on lncRNA-driven cancer phenotypes [122]. In addition, berberine-mediated modulation of the lncRNAs CCDC18-AS1 and TBILA appears to be involved in its inhibitory effects on esophageal cancer progression and in improved patient prognosis [123].

#### 4.1.4. Convergent Signaling Axes Regulated by Polyphenol-Sensitive LncRNAs

Despite structural diversity, polyphenols converge on a limited set of oncogenic signaling pathways through lncRNA-mediated regulation. These include NF-κB, PI3K-AKT-mTOR, and Wnt/β-catenin signaling axes. LncRNAs such as HOTAIR, MALAT1, NEAT1, and H19 serve as regulatory nodes within these pathways, affecting transcriptional output, signal amplification, and pathway crosstalk [20,21,124].

Polyphenol-induced repression of NF-κB-associated lncRNAs attenuates chronic inflammatory signaling within the tumor microenvironment. Modulation of PI3K-AKT-linked lncRNAs alters metabolic adaptation and survival signaling, while regulation of Wnt/β-catenin-associated lncRNAs limits EMT and stemness programs [125,126,127]. Importantly, these pathways are interconnected through lncRNA-mediated feedback loops, allowing dietary compounds to exert systems-level regulatory effects rather than isolated molecular inhibition [128].

Together, these findings establish polyphenols and flavonoids as modulators of lncRNA-driven cancer networks and provide a mechanistic rationale for their inclusion in lncRNA-guided chemoprevention strategies.

### 4.2. Omega-3 and Omega-6 Fatty Acids: LncRNA-Mediated Control of Inflammation, Metabolism, and Tumor Progression

PUFAs, specifically omega-3 (ω-3) and omega-6 (ω-6) fatty acids, play critical roles in regulating inflammation, membrane dynamics, and metabolic signaling, processes that are closely associated with cancer development and progression. Beyond their established effects on lipid metabolism and eicosanoid synthesis, increasing evidence indicates that PUFAs modulate DNA methylation and lncRNA expression, thereby influencing cancer-relevant transcriptional and epigenetic programs [129,130]. These findings position fatty acids as potent metabolic regulators of lncRNA-driven cancer pathways.

The ω-6 PUFAs, through their pro-inflammatory derivatives, are generally associated with tumor-promoting microenvironments, whereas ω-3 PUFAs, specifically eicosapentaenoic acid (EPA) and docosahexaenoic acid (DHA), exert anti-inflammatory and anti-tumorigenic effects. A key factor is that the ratio between ω-6 and ω-3, as a lower ratio is strongly linked to reduced breast cancer risk and fewer chronic diseases [131]. Increasingly, these opposing biological outcomes are understood to be mediated, at least in part, by differential regulation of lncRNA networks that govern inflammation, metabolic adaptation, and cellular plasticity.

Transcriptomic analyses of human visceral adipocytes provide direct support for this concept, demonstrating that individual PUFAs elicit distinct and context-dependent lncRNA responses [130]. Adipocytes isolated from normal-weight individuals exposed to arachidonic acid (AA), an ω-6 fatty acid, display upregulation of several oncogenic lncRNAs, including LINC01106, SNHG11, SNHG17, and TRIM52-AS1, alongside downregulation of tumor-suppressive lncRNAs such as MAGI2-AS3 and NR2F1-AS1. Similarly, AA treatment of adipocytes from colorectal cancer patients induces expression of the oncogenic lncRNA, MSC-AS1. Network-based analyses also indicate that AA-responsive lncRNAs serve as regulatory hubs, connecting RNA-binding proteins with larger transcriptional programs that promote inflammation and support tumor growth within fat tissue. These results are indicative of the fact that AA treatment is linked to colorectal cancer development, adipose tissue problems, or growth factor–driven pathways such as MAPK, EGFR, and NOTCH signaling.

Collectively, these data indicate that AA-responsive lncRNAs act as central regulatory nodes linking inflammatory signaling, RNA-binding protein networks, and transcriptional programs that sustain tumor-promoting adipose tissue microenvironments. Together, these findings underscore the tumor-promoting and tumor-sustaining roles of ω-6 fatty acids, exemplified here by AA [130].

In contrast, the ω-3 PUFA, DHA, selectively modulates lncRNAs linked to anti-inflammatory signaling and metabolic balance. DHA treatment decreases LUCAT1, a well-known oncogenic lncRNA previously shown to be elevated in obesity and colorectal cancer, and suppresses PSMG3-AS1, which has been associated with gastrointestinal cancers. Notably, this ω-3-induced lncRNA response is significantly diminished in adipocytes from obese individuals and colorectal cancer patients, highlighting disease-associated impairments in nutrient responsiveness [130].

**Figure 3:**
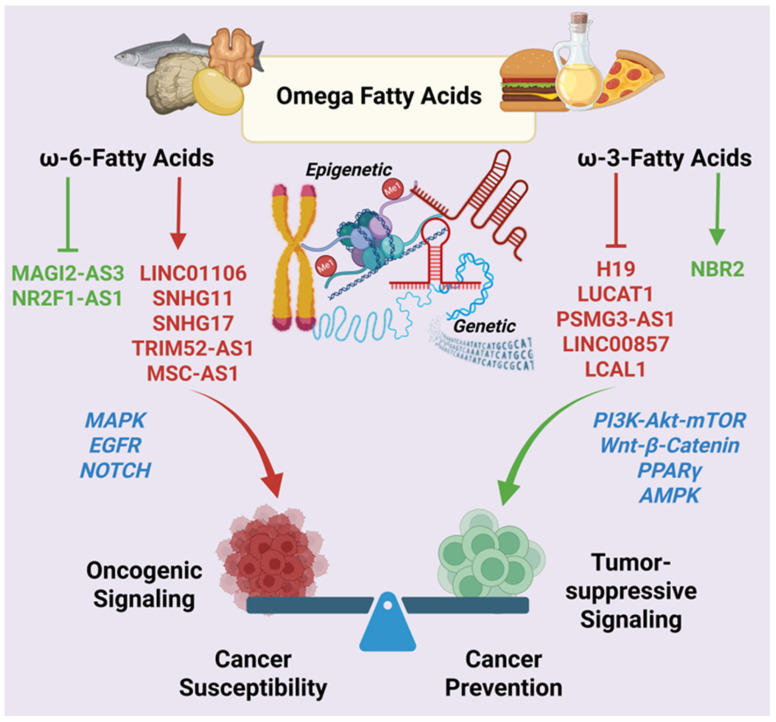
Differential regulation of lncRNA networks by omega-3 (ω-3) and omega-6 (ω-6) fatty acids. This schematic illustrates how distinct classes of dietary ω fatty acids modulate cancer-relevant lncRNA programs and signaling pathways. ω-3 fatty acids suppress multiple oncogenic lncRNAs (e.g., H19, LINC00857, LCAL1, LUCAT1, and PSMG3-AS1) while inducing tumor-suppressive lncRNAs such as NBR2. These changes converge on key pathways governing inflammation, metabolism, and growth control, including PI3K–AKT–mTOR, Wnt/β-Catenin, PPARγ, and AMPK signaling. In contrast, ω-6 fatty acids are associated with the induction of oncogenic lncRNAs (e.g., LINC01106, SNHG11, SNHG17, TRIM52-AS1, and MSC-AS1) and suppression of tumor suppressive lncRNAs such as MAGI2-AS3 and NR2F1-AS1, that preferentially engage MAPK, EGFR, and NOTCH signaling cascades. The balance between ω-3 and ω-6 fatty acids influences adipose tissue inflammation, metabolic reprogramming, and cancer-associated signaling through lncRNA-centered regulatory networks. Oncogenic lncRNAs are indicated in red, whereas tumor-suppressive lncRNAs are shown in green. Reported effects are context-dependent and influenced by tissue type, metabolic state, and disease status. For clarity, individual lncRNAs are depicted according to their predominant reported functional role.

Taken together, these findings highlight lncRNAs as important molecular intermediates through which ω-3 and ω-6 PUFAs differentially shape adipose tissue inflammation, metabolic reprogramming, and tumor-associated signaling (Figure 3). This lncRNA-centered nutrient responsiveness offers a plausible mechanistic link between dietary fatty acid composition, adipose tissue dysfunction, and cancer progression (Figure 3).

#### 4.2.1. EPA and DHA: Repression of Pro-Tumorigenic LncRNAs and Epigenetic Remodeling

ω-3 PUFAs such as EPA and DHA have been shown to suppress the expression of the imprinted oncogenic lncRNA H19 [132]. Downregulation of H19 by EPA and DHA has been associated with reduced proliferative signaling, decreased invasive capacity, and altered DNA methylation patterns in cancer cells [133,134]. In addition, H19 is known to interact with epigenetic regulators such as DNMT1, linking fatty acid availability to locus-specific DNA methylation and transcriptional repression. Therefore, ω-3 PUFA-induced modulation of H19 may influence broader epigenetic landscapes that govern inflammatory signaling and metabolic gene expression [135,136]. At the signaling level, EPA and DHA modulation of H19 could impact diverse oncogenic pathways including PI3K-AKT and Wnt/β-catenin signaling pathways [130,137].

#### 4.2.2. LncRNAs in Fatty Acid-Regulated Inflammation and Macrophage Polarization

Chronic inflammation is a key driver of tumor initiation and progression, and macrophages play a central role in shaping inflammatory tumor microenvironments. LncRNAs have emerged as critical regulators of macrophage polarization, influencing the balance between pro-inflammatory (M1-like) and immunosuppressive (M2-like) states [138]. Several inflammation-associated lncRNAs respond to PUFA availability, linking lipid metabolism to immune regulation [138]. The lncRNAs such as H19, SNHG11, SNHG17, MSC-AS1, and LUCAT1 have been implicated in various facets of inflammatory responses and cytokine production [139,140,141,142,143,144]. Since ω-3 as well as ω-6 fatty acids have been shown to modulate the expression of these lncRNAs [130], both of these families of fatty acids contribute to tumor-immune microenvironments underlying tumorigenesis and tumor growth.

#### 4.2.3. LncRNA Control of Lipid-Sensitive Transcriptional Regulators: PPARγ and AMPK

Beyond inflammation, ω-3 fatty acids influence cancer metabolism through lncRNA-dependent modulation of lipid-sensitive transcriptional regulators [145]. The nuclear receptor, peroxisome proliferator-activated receptor gamma (PPARγ) and the energy sensor, AMP-activated protein kinase (AMPK) represent key nodes in metabolic adaptation, lipid handling, and tumor growth control [146,147].

The lncRNA lnc-HC negatively regulates PPARγ expression by modulating miRNA availability, thereby influencing lipid droplet formation and hepatic lipid accumulation. Changes in fatty acid composition, including ω-3 PUFA enrichment, alter lnc-HC expression and downstream PPARγ activity, linking dietary lipids to transcriptional control of metabolism [148].

AMPK signaling is also tightly regulated by lncRNAs. The lncRNA NBR2 directly interacts with the AMPKα subunit, enhancing AMPK activation under metabolic stress and suppressing tumorigenesis [149]. Conversely, lncRNAs such as H19, LINC00857, and LCAL1 modulate AMPK phosphorylation status and downstream mTOR signaling, influencing autophagy, proliferation, and metabolic rewiring [150]. ω-3 fatty acids, by altering cellular energy balance and lipid flux, indirectly shape these lncRNA-AMPK circuits, reinforcing growth-restrictive metabolic states [151].

These findings highlight fatty acids as metabolic regulators capable of exerting systems-level control over cancer-relevant lncRNA networks. Importantly, the lncRNA-mediated effects of ω-3 fatty acids complement those observed for polyphenols and flavonoids, reinforcing the concept that diverse dietary bioactive compounds converge on shared lncRNA-centered regulatory architectures. This convergence provides a strong mechanistic rationale for incorporating fatty acid–lncRNA interactions into precision nutrition-guided cancer chemoprevention strategies.

### 4.3. Niacin, NAD^+^ Metabolism, and Sirtuin–LncRNA Axes in Cancer Regulation

Cellular NAD^+^ metabolism occupies a central position at the interface of energy homeostasis, redox balance, chromatin regulation, and stress adaptation, processes that are frequently dysregulated in cancer [152]. Niacin (vitamin B3), as a dietary precursor of NAD^+^, influences intracellular NAD^+^ pools and thereby regulates the activity of NAD^+^-dependent enzymes, including sirtuins and poly (ADP-ribose) polymerases (PARPs) [153]. Emerging evidence indicates that lncRNAs actively participate in these NAD^+^-sensitive regulatory circuits, positioning them as key intermediaries linking niacin availability to cancer-relevant transcriptional and epigenetic programs [8,154]. This bidirectional relationship places lncRNAs at the center of nutrient-sensitive epigenetic regulation in cancer cells [155].

Unlike static metabolic cofactors, NAD^+^ functions as a dynamic signaling molecule that integrates nutrient availability with chromatin remodeling and transcriptional control [156]. LncRNAs respond to fluctuations in NAD^+^ levels and, in turn, regulate the expression, localization, and activity of NAD^+^-dependent enzymes. In this context, NAD+-dependent class III protein deacetylases, the sirtuins, play a critical role in the regulation of tumor genesis and tumor progression [157].

#### 4.3.1. Sirtuin–LncRNA Interactions in Chromatin Remodeling and Tumor Suppression

Sirtuins (SIRT1–SIRT7) are NAD^+^-dependent deacetylases that regulate histone modification, transcription factor activity, mitochondrial function, and genome stability [158]. Several lncRNAs directly or indirectly modulate sirtuin expression and function, thereby influencing cancer cell fate.

In colorectal cancer, tumor-suppressive lncRNA, GAS5 is downregulated; however, it acts as a ceRNA, sequesters miR-34a, resulting in the upregulation of SIRT1, which suppresses macroautophagy, and promotes apoptosis, thereby inhibiting cell survival [159]. Similar miRNA-sequestration was used by several lncRNAs such as lincRNA-p21, to control the expression of SIRT1 in a variety of cancers. The SIRT1–lncRNA axis is a promising target for cancer therapy because of these interactions, which affect important processes like autophagy, apoptosis, chemoresistance, and metabolic reprogramming [160,161]. Increased NAD^+^ levels, driven by niacin supplementation or enhanced salvage pathway activity, can promote SIRT1 activity, leading to deacetylation of p53, FOXO transcription factors, and NF-κB subunits [162,163]. Higher SIRT1 activity reduces the H3K18la on H19 promoter, to downregulate this oncogenic lncRNA and attenuate gastric cancer progression [164]. These results indirectly link the regulation of lncRNAs such as H19 by niacin supplementation.

Conversely, tumor-suppressive lncRNAs such as MEG3 enhance p53 stability and transcriptional activity, an effect that is reinforced by SIRT1-mediated chromatin remodeling under conditions of adequate NAD^+^ availability [165]. These findings highlight how niacin-sensitive sirtuin activity intersects with lncRNA networks to regulate tumor suppressor pathways.

#### 4.3.2. NAD^+^ Salvage Pathway, NAMPT, and lncRNA Control of Metabolic Plasticity

The NAD^+^ salvage pathway, primarily driven by nicotinamide phosphoribosyl transferase (NAMPT), is often upregulated in cancer cells to support high metabolic and biosynthetic needs [166]. This sheds light on the context-dependent regulatory roles of niacin supplementation as it enriches the NAD^+^ pool through NAMPT, thereby promoting oncogenic signaling. LncRNAs have become key regulators of NAMPT expression and activity, thus affecting NAD^+^ availability and downstream signaling.

The oncogenic lncRNA UCA1 promotes glycolytic flux and metabolic reprogramming in part by enhancing NAMPT expression and stabilizing NAD^+^ pools, supporting sustained proliferation and therapy resistance [167,168]. Such lncRNA-driven circuits reinforce the metabolic adaptability of cancer cells under nutrient stress.

In contrast, lncRNAs such as NBR2, which activate AMPK signaling during stress, indirectly suppress excessive NAD^+^ consumption by limiting anabolic growth and mTOR signaling [149]. Through this mechanism, NBR2 acts as a metabolic checkpoint that counterbalances NAD^+^-driven oncogenic programs. Niacin availability, affected by NAD^+^ synthesis, modulates the balance between these competing lncRNA-regulated metabolic states [169].

#### 4.3.3. PARP Activity, DNA Damage Responses, and lncRNA Regulation

NAD^+^ also serves as an essential substrate for PARPs, which regulate DNA damage responses and genome stability. Excessive PARP activation can deplete NAD^+^ pools, leading to metabolic collapse, whereas controlled PARP activity supports DNA repair and cell survival [170]. Nicotinamide, enriched by niacin supplementation, are being explored as both promoters and inhibitors of PARP activity in a context- and concentration-dependent manner [171,172]. Studies have shown that augmenting NAD^+^ pools have enhanced lncRNA-mediated PARP activity. Upon enrichment of NAD^+^ pool through NMNAT1 supplementation, the lncRNA EGFR-AS1 has been shown to act as a scaffold for PARP1 complex and promote DNA repair through the modulation of PARP-dependent pathways [173]. In addition, other lncRNAs, including LIP, scaffold DNA repair complexes involving PARP1 and influence base-excision repair efficiency, intersecting with PARP-dependent repair mechanisms [174].

In contrast, recent studies have also explored niacin supplementation or nicotinamide as PARP inhibitor. Nicotinamide was reported to be used as a post-chemotherapy maintenance treatment for BRCA-mutated ovarian cancers [175]. Niacin-mediated modulation of NAD^+^ levels therefore impacts not only metabolic regulation but also lncRNA-governed DNA damage responses relevant to cancer susceptibility and progression [171].

Collectively, niacin and NAD^+^ metabolism influence cancer biology through tightly coupled lncRNA-dependent regulatory networks that integrate chromatin remodeling, metabolic adaptation, inflammatory signaling, and genome stability [51]. By modulating sirtuin activity, NAD^+^ salvage pathways, and PARP-dependent DNA repair, niacin-sensitive lncRNA circuits shape transcriptional and epigenetic landscapes that determine cancer cell fate.

### 4.4. Folate, One-Carbon Metabolism, and LncRNA-Driven Epigenetic Regulation

Folate metabolism plays a crucial role in one-carbon transfer reactions that support nucleotide biosynthesis, DNA methylation, and chromatin remodeling. Also, vitamins B2, B6, and B12 act as cofactors in one-carbon metabolism, facilitating folate cycling and methionine regeneration. This process produces S-adenosylmethionine (SAM), the primary methyl donor for epigenetic and metabolic methylation reactions. Homocysteine is either recycled to methionine or converted to cysteine, with dietary betaine serving as an additional methyl donor [176,177]. Disruption of folate homeostasis alters epigenetic landscapes and genome stability, processes that are intimately linked to carcinogenesis. Increasing evidence indicates that lncRNAs function as sensitive regulators as well as targets of folate-dependent epigenetic mechanisms, connecting dietary folate intake to lncRNA-driven cancer pathways [5].

Through its role in generating SAM, folate influences DNA and histone methylation patterns that govern lncRNA transcription. Conversely, lncRNAs modulate the expression and activity of enzymes involved in one-carbon metabolism, creating bidirectional regulatory loops that shape epigenetic states relevant to cancer development [178].

#### 4.4.1. Vitamin B-Dependent DNA Methylation and LncRNA Expression

Altered folate levels influence the methylation status of lncRNA promoters, leading to changes in lncRNA expression that affect cancer susceptibility. Hypomethylation-driven overexpression of oncogenic lncRNAs such as HOTAIR, H19, and UCA1 has been associated with enhanced proliferation, EMT, and metastatic potential [5]. Folate deficiency exacerbates these effects by reducing methyl donor availability, thereby promoting permissive chromatin states that favor oncogenic lncRNA activation.

Folate deficiency is associated with DNA strand breaks, impaired DNA repair, and chromosomal instability, all of which contribute to carcinogenesis [179]. Though there is no direct evidence of folate deficiency regulating lncRNAs mediating the DNA damage repair, further studies are required to unravel these aspects. However, lncRNAs such as NORAD and LINP1, which are involved in genome maintenance and DNA repair pathway coordination, are sensitive to epigenetic and metabolic perturbations that possibly could be linked to folate status [180,181]. Altered expression of these lncRNAs under folate-deficient conditions compromises genome integrity and promotes accumulation of oncogenic mutations.

Importantly, excessive folate supplementation in established tumors may also have unintended consequences by supporting nucleotide biosynthesis and proliferation. This duality underscores the context-dependent nature of folate–lncRNA interactions and reinforces the need for lncRNA-guided stratification when considering folate-based interventions for cancer prevention [169].

In addition, metabolic pathways of certain vitamins have been reported to involve the upregulation of certain lncRNAs that promote cancer progression. Bioinformatic evidence suggests that the riboflavin transporter SLC52A2 is embedded within lncRNA-centered regulatory networks in hepatocellular carcinoma, notably involving the THUMPD3-AS1/hsa-miR-139-5p/SLC52A2 axis, highlighting a potential connection between riboflavin metabolism, lncRNA regulation, and cancer-related epigenetic processes [182]. Moreover, vitamin B6 metabolism, via the enzyme PNPO, is linked to breast invasive ductal carcinoma (IDC) development. PNPO is overexpressed in ovarian and breast cancer, promoting tumor progression, and positively correlates with the lncRNA MALAT1, which inversely correlates with miR-216b-5p. This suggests that the MALAT1/miR-216b-5p/PNPO axis plays a key role in IDC and it could be a therapeutic target [183]. Vitamin B12 also affects lncRNAs, particularly upregulation of MALAT1, in mouse N2A neuroblastoma cells [169,184].

#### 4.4.2. LncRNAs as Regulators of One-Carbon Metabolic Enzymes

Beyond being epigenetic targets, lncRNAs actively regulate enzymes involved in folate and one-carbon metabolism. The lncRNA HULC has been shown to influence methionine cycle enzymes and SAM availability, indirectly affecting global methylation capacity and transcriptional regulation [185]. Similarly, MALAT1 and NEAT1 modulate chromatin-associated metabolic enzymes, linking lncRNA expression to metabolic control of epigenetic states [186].

These regulatory interactions establish feedback loops in which folate availability shapes lncRNA expression, and lncRNAs, in turn, influence the efficiency and fidelity of one-carbon metabolic processes. Disruption of these loops can promote epigenetic instability and facilitate malignant transformation. Together, folate-dependent one-carbon metabolism and lncRNA-mediated epigenetic regulation form an interconnected network that influences cancer initiation and progression. By modulating DNA methylation, chromatin structure, and genome stability through lncRNA-dependent mechanisms, folate availability shapes cancer-relevant transcriptional landscapes.

**Figure 4:**
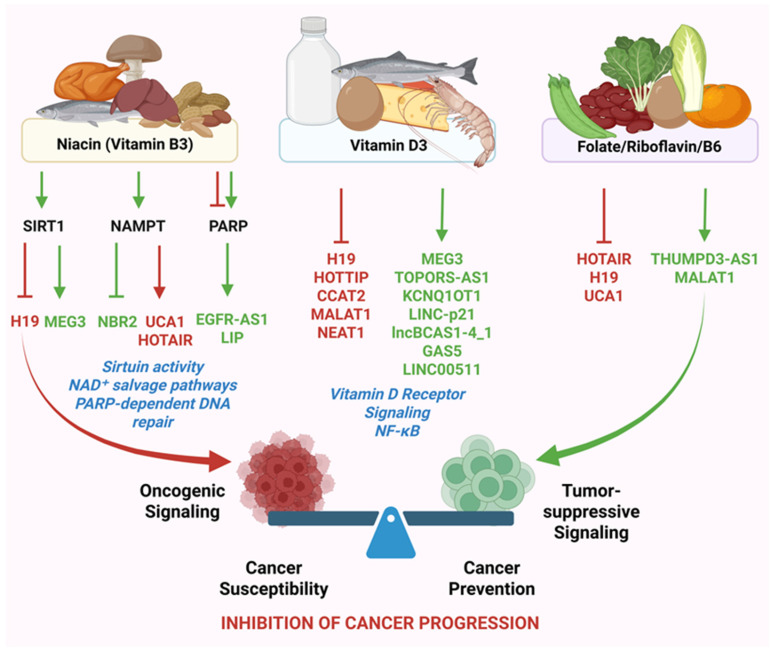
Regulation of cancer-associated lncRNAs by niacin (vitamin B3), vitamin D3, folate, riboflavin and vitamin B6. This figure summarizes how vitamins from the diet shape cancer-relevant lncRNA programs through complementary metabolic and epigenetic routes. Niacin acts primarily through NAD^+^-dependent processes, including sirtuin (SIRT1) signaling, NAMPT-driven NAD^+^ salvage, and PARP-mediated DNA repair. Through these pathways, niacin is linked to reduced expression of oncogenic lncRNAs such as H19, UCA1, and HOTAIR, alongside induction of tumor-suppressive lncRNAs including MEG3, NBR2, EGFR-AS1, and LIP, consistent with enhanced genome maintenance and stress resilience. Vitamin D3 is associated with repression of oncogenic lncRNAs (H19, HOTTIP, CCAT2, MALAT1 and NEAT1) and induction of tumor-suppressive lncRNAs (MEG3, TOPORS-AS1, KCNQ1OT1, LINC-p21, lncBCAS1-4_1, GAS5 and LINC00511). Folate influences lncRNA expression through its role in one-carbon metabolism and DNA methylation. Adequate folate/riboflavin/vitamin B6 availability is associated with repression of oncogenic lncRNAs (HOTAIR, H19, UCA1) and increased expression of tumor-suppressive lncRNAs such as THUMPD3-AS1 and MALAT1. Together, these vitamin-responsive lncRNA networks converge on pathways that favor genomic stability, metabolic balance, and restraint of cancer progression. Red text denotes oncogenic lncRNAs, whereas green text denotes tumor-suppressive lncRNAs. Because vitamin effects can be concentration- and context-dependent, lncRNAs are depicted according to their predominant reported regulatory outcome.

These findings position folate not merely as a nutritional requirement but as an epigenetic regulator whose effects on cancer risk are mediated through lncRNA-centered networks. Incorporating folate-lncRNA interactions into precision nutrition frameworks may help distinguish protective versus permissive epigenetic states and guide more informed chemopreventive strategies. Figure 4 summarizes the emerging roles of niacin (vitamin B3) and folate in reshaping cancer-associated lncRNA networks through metabolic and epigenetic regulatory pathways.

Beyond direct effects on cancer cells, several B vitamins exert measurable immunomodulatory effects that may indirectly influence lncRNA-regulated inflammatory and tumor-permissive environments. In human immune cells, vitamins E, K, B5, B6, and B9 have been shown to differentially modulate immune cell subsets and activation states, as assessed by flow cytometry, highlighting the capacity of micronutrients to reshape immune contexts relevant to cancer initiation and progression [187].

### 4.5. Vitamin D–LncRNA Networks in Cancer Regulation

Vitamin D, through its active metabolite 1,25-dihydroxyvitamin D_3_, exerts pleiotropic effects on cellular differentiation, immune regulation, and growth control [188]. These effects are primarily mediated by the vitamin D receptor (VDR), a ligand-activated nuclear receptor that functions as a transcriptional regulator. Beyond classical protein-coding targets, accumulating evidence indicates that VDR signaling intersects extensively with lncRNA networks, positioning lncRNAs as critical downstream effectors of vitamin D-dependent cancer regulation [169].

VDR binding to vitamin D response elements within promoter or enhancer regions influences the transcription of multiple lncRNAs involved in cell cycle control, differentiation, and immune modulation. In the cancer context, vitamin D signaling has been linked to the repression of oncogenic lncRNAs and induction of tumor-suppressive lncRNAs, thereby reshaping transcriptional programs linked to cell proliferation and invasion [189]. In particular, vitamin D3 has been shown to inhibit glycolysis and lactate production in colorectal cancer by inducing the expression of MEG3 [190]. These effects underscore a direct epigenetic interface between vitamin D availability and lncRNA-driven cancer pathways.

#### 4.5.1. VDR-Regulated LncRNAs in Proliferation and Differentiation

Several lncRNAs are transcriptionally regulated by VDR signaling and contribute to vitamin D-mediated growth restraints. The lncRNA MEG3, a well-established tumor suppressor that enhances p53 activity and represses oncogenic transcriptional programs, is positively regulated by vitamin D in multiple epithelial cancer models [191]. Upregulation of MEG3 in response to vitamin D correlates with reduced proliferation and increased differentiation, reinforcing its role as a mediator of VDR-dependent tumor suppression. A tumor suppressive lncRNA, TOPORS-AS1, which inhibits the Wnt/β-catenin pathway in ovarian cancer, is upregulated after the activation of VDR by vitamin D. TOPORS-AS1 expression is correlated with better prognosis in ovarian cancer [192]. In another study, VDR knockout in keratinocytes disrupted the transcriptomic balance, upregulating oncogenic lncRNAs such as H19 and HOTTIP and downregulating tumor-suppressive lncRNAs such as KCNQ1OT1 and LINC-p21, highlighting the protective of VDR towards the development of skin cancer [193].

Suppression of CCAT2 in ovarian cancer cells (SKOV3 and OVCAR3) following treatment with calcitriol (1,25-dihydroxyvitamin D3) restricts its binding with TCF4 and consequently blocks downstream expression of c-Myc. This results in restricted proliferation, migration, and invasion in ovarian cancer cells [194]. Vitamin D signaling and EMT suppression are significantly associated with 1α,25(OH)_2_D_3_-induced lncBCAS1-4_1 upregulation in the ovarian cancer cell line, SKOV3. Suppression of this lncRNA promotes cellular proliferation and migration in ovarian cancer [195]. Vitamin D signaling also represses oncogenic lncRNAs such as H19 and MALAT1, which are implicated in EMT, metastasis, and therapy resistance. Suppression of these lncRNAs attenuates proliferative signaling and disrupts transcriptional programs associated with cellular plasticity [187]. These findings suggest that vitamin D-VDR activity modulates a balance between oncogenic and tumor-suppressive lncRNA networks that influence cancer progression.

#### 4.5.2. Vitamin D, Immune Regulation, and LncRNA-Mediated Tumor Microenvironment Control

Vitamin D is a potent immunomodulator that shapes both innate and adaptive immune responses. LncRNAs have emerged as key regulators of immune cell differentiation, cytokine signaling, and immune checkpoint expression, providing a mechanistic link between vitamin D signaling and tumor immune surveillance. The lncRNA NEAT1 plays a central role in inflammatory signaling and immune cell activation. Vitamin D-mediated suppression of NEAT1 has been associated with reduced NF-κB activation and attenuation of pro-tumorigenic inflammatory signaling within the tumor microenvironment [189]. Additional immune-related lncRNAs, including LINC00511 and GAS5, respond to vitamin D signaling and modulate T-cell function, macrophage polarization, and cytokine production in breast cancer [196]. Through these mechanisms, vitamin D-regulated lncRNA networks contribute to immune contexts that are less permissive to tumor growth.

Collectively, vitamin D signaling engages lncRNA-dependent regulatory circuits that constrain proliferation, promote differentiation, and modulate tumor-associated inflammation. By acting through VDR-regulated lncRNAs, vitamin D influences cancer biology at the transcriptional and epigenetic levels rather than through isolated signaling pathways. These findings provide a mechanistic rationale for considering vitamin D status within lncRNA-guided frameworks for cancer risk modulation and chemoprevention.

### 4.6. Probiotics and Postbiotics in LncRNA-Mediated Prevention of Colorectal Carcinogenesis

Dietary elements, including amino acids, carbohydrates, lipids, and fibers (acting as prebiotics) along with polyphenols, influence the growth and metabolism of the microbial flora that inhabit the gut, which in turn influence with their metabolites (postbiotics) the integrity and homeostasis of intestinal tissue [71,197]. Alterations in the microbiota composition (dysbiosis) may lead to intestinal inflammatory diseases (such as inflammatory bowel disease and Crohn’s disease) and favor colorectal carcinogenesis [198,199]. Interestingly, CARINH, a lncRNA transcribed from a locus associated with increased risk of inflammatory bowel disease, has been shown to protect the intestinal mucosa by maintaining gut eubiosis [200]. Certain bacteria have been shown to promote colorectal carcinogenesis and progression by modulating the expression of lncRNAs in intestinal cells [201]. For instance, *Fusobacterium nucleatum* has been shown to promote the metastatization of colorectal cancer through inducing the expression of two onco-lncRNAs EVADR and KRT7-AS [202,203]

On the contrary, certain lactobacillus strains, with their metabolites (particularly the short-chain fatty acids butyrate, propionate and acetate), have been shown to dampen inflammation, revert the macrophage M2 into M1 phenotype, stimulate the immune response and restore intestinal epithelial barrier, thus providing preventive and therapeutic benefits in colorectal cancer [199,204,205,206]. Among the variety of mechanisms involved in such anticancer activity by probiotics and postbiotics is their ability to modulate the expression of tumor suppressor lncRNAs [201,207,208]. A recent placebo-controlled clinical trial demonstrated that lncRNAs may play a key role in mediating the anticancer effects of beneficial probiotics. In colorectal cancer patients undergoing chemo-radiotherapy, consumption of probiotic *Lactobacillus acidophilus* for 13 weeks led to substantial downregulation of the expression of eleven onco-lncRNAs (including PVT1, HOTAIR, MALAT1, UCA1, CCAT1, CRNDE, XLOC_006844, LOC152578, XLOC-000303, and BCAR4) and the upregulation of the expression of the tumor suppressor lncRNA, LincRNA-P21 [209].

Only a limited number of studies have directly examined the relationship between postbiotics and lncRNA regulation. Butyrate, a short-chain fatty acid predominantly generated through microbial fermentation of dietary fiber, has been shown to induce the expression of LOH12CR2, a lncRNA that suppresses colorectal tumorigenesis by destabilizing the mRNA of the mitotic regulator SPC24 via METTL14-mediated N6-methyladenosine modification [210]. In a separate study, exposure of colorectal cancer cells to butyrate at concentrations that inhibit proliferation and invasion resulted in the differential regulation of 30 lncRNAs, with 21 upregulated and 9 downregulated. Notably, nine of these lncRNAs (six upregulated and three downregulated) participated in ceRNA networks involving miRNAs and mRNAs linked to colorectal cancer progression and prognosis [211].

**Figure 5:**
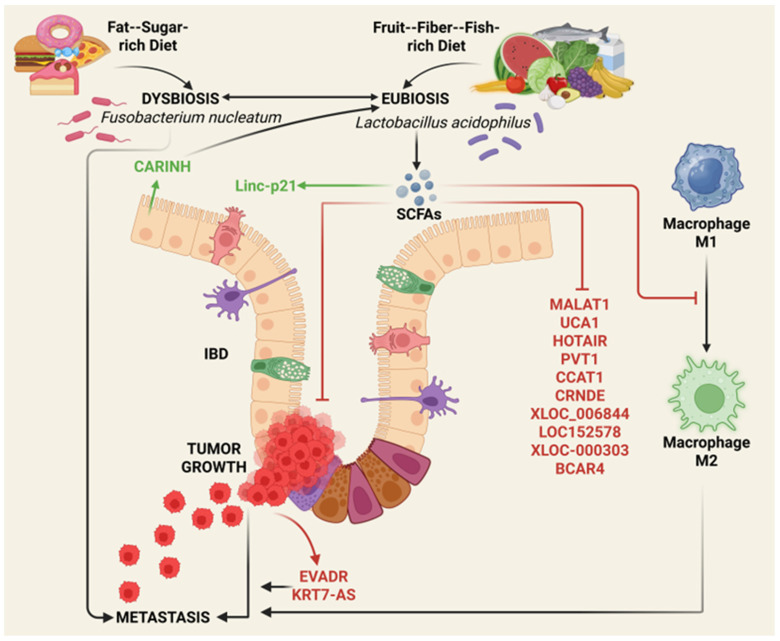
lncRNA-mediated integration of diet, gut microbiota, and colorectal cancer progression. This schematic illustrates how dietary patterns shape gut microbial composition and downstream lncRNA-regulated pathways that influence intestinal inflammation and colorectal cancer progression. A fat- and sugar-rich diet promotes intestinal dysbiosis, characterized by enrichment of pro-inflammatory bacteria such as *Fusobacterium nucleatum*, leading to chronic inflammation, inflammatory bowel disease (IBD), and a tumor-promoting microenvironment. Dysbiosis-associated bacteria induce oncogenic lncRNAs, including EVADR and KRT7-AS, which facilitate tumor growth, immune evasion, and metastatic progression. In contrast, a diet rich in fruits, fiber, and fish supports gut eubiosis, characterized by short-chain fatty acid (SCFA)-producing bacteria such as Lactobacillus acidophilus. SCFAs, particularly butyrate, suppress oncogenic lncRNAs including MALAT1, UCA1, PVT1, and HOTAIR, thereby restraining malignant signaling. The intestinal epithelial lncRNA CARINH contributes to maintenance of eubiosis and mucosal homeostasis, limiting inflammation and colorectal tumorigenesis. Collectively, this axis highlights lncRNAs as molecular mediators through which diet–microbiota interactions modulate intestinal inflammation, tumor growth, and metastatic potential. Color coding distinguishes lncRNA function, with red indicating oncogenic lncRNAs and green indicating tumor-suppressive lncRNAs. While emerging evidence supports these links, the specific lncRNAs mediating probiotic and postbiotic antitumor effects remain an active area of investigation.

These interconnected relationships between diet, gut microbial composition, microbial metabolites, and lncRNA-mediated regulation of intestinal homeostasis and colorectal carcinogenesis are schematically summarized in Figure 5. While this framework highlights emerging mechanistic links, substantial work remains to define the specific lncRNAs that mediate the antitumor effects of probiotics and postbiotics.

### 4.7. Essential Trace Elements-LncRNA Axis in Cancer

Essential trace elements such as selenium and zinc play indispensable roles in maintaining cellular redox balance, genome integrity, and adaptive stress responses, biological processes that are frequently disrupted during carcinogenesis [212,213,214,215]. Beyond their classical functions as enzymatic cofactors, emerging evidence indicates that selenium and zinc modulate the expression of lncRNAs, directly and/or indirectly, thereby linking micronutrient availability to cancer-relevant transcriptional and epigenetic programs [216,217].

Primarily, these trace elements modulate lncRNA expression by altering redox-sensitive transcription factors and epigenetic regulators, rather than by direct interaction with lncRNA loci. Although the underlying mechanism is not known, it has been shown that sodium selenite inhibits the malignant progression of gastric cancer through the downregulation of lncRNA, HOXB-AS1 [218].

Selenium is incorporated into a specialized class of selenoproteins [219], including glutathione peroxidases and thioredoxin reductases, which are central to antioxidant defense and redox signaling. Through these systems, selenium availability influences cellular responses to oxidative stress, inflammation, and DNA damage, conditions known to regulate the expression profiles of lncRNAs in cancer [220]. Consistent with this role, selenium nanoparticles have been shown to overcome sorafenib resistance in thioacetamide-induced hepatocellular carcinoma in rats by downregulating the lncRNA AF085935. Suppression of AF085935 reduces glypican-3 (GPC3) expression, leading to decreased angiogenesis and metastasis and enhanced apoptosis [221]. In another study, selenium deficiency–induced exudative diathesis in broiler chicks was associated with oxidative vascular damage and dysregulation of 635 lncRNAs, many of which contributed to redox-sensitive stress responses, further underscoring the role of selenium in pathways associated with cancer progression [222].

Zinc, similarly, functions as a structural and catalytic cofactor for a wide range of transcription factors and chromatin-associated proteins, including p53, NF-κB, and zinc-finger regulators [223]. Through these roles, zinc availability indirectly shapes lncRNA expression programs involved in DNA damage responses, inflammatory signaling, and cell survival.

Collectively, these findings position redox-sensitive, selenium- and zinc-responsive lncRNAs as molecular intermediaries linking micronutrient availability to redox control and cancer susceptibility. Incorporating trace element-lncRNA interactions into precision nutrition frameworks may therefore provide additional leverage points for redox-oriented cancer prevention strategies, complementing broader dietary and metabolic interventions discussed elsewhere in this review.

## 5. Integrative and Systems-Level Approaches to Decode Nutrient–LncRNA–Cancer Networks

### 5.1. Rationale for Systems-Level Analysis of Nutrient-Responsive LncRNA Regulation

**Figure 6:**
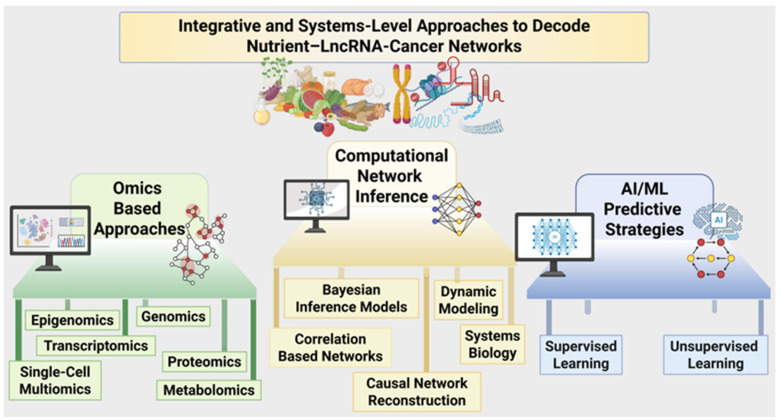
AI and ML paradigm for decoding nutrient–lncRNA–cancer regulatory networks. This schematic illustrates an emerging framework for systematically resolving how nutritional inputs from diet shape cancer biology through lncRNA-centered regulatory networks. Multi-omics and single-cell profiling provide foundational molecular layers to capture nutrient-responsive lncRNA dynamics across genetic, epigenetic, transcriptional, proteomic, and metabolic dimensions. These data are integrated using computational network inference strategies—including correlation-based modeling, Bayesian and causal network reconstruction, dynamic modeling, and systems biology approaches—to identify regulatory hierarchies, context-specific control nodes, and emergent network behavior. AI/ML strategies further extend this paradigm by enabling data integration at scale, pattern discovery, and predictive modeling of nutrient–lncRNA–pathway interactions. These approaches are intended as decision-support and discovery tools rather than validated clinical interventions. Together, they define a forward-looking systems framework to guide mechanistic discovery, biomarker development, and precision nutrition–based cancer prevention strategies.

The regulatory role of lncRNAs in cancer biology is inherently network-based rather than linear. Individual lncRNAs interact with chromatin modifiers, transcription factors, RNA-binding proteins, miRNAs, and signaling pathways in highly context-dependent ways. When combined with nutritional inputs, which simultaneously influence metabolism, redox balance, inflammatory signaling, and epigenetic regulation, the resulting regulatory landscape becomes multidimensional and non-linear. Therefore, traditional reductionist approaches are inadequate to fully understand how nutrient-responsive lncRNA networks influence cancer susceptibility and progression. Figure 6 outlines a systems-level paradigm that unifies multi-omics profiling, computational network inference, and AI/machine learning (ML)-driven prediction to enable mechanistic interpretation and translational insight into nutrient–lncRNA–cancer networks.

Systems biology offers a conceptual and computational framework to integrate complex interactions across molecular layers. By modeling lncRNAs as dynamic nodes within regulatory networks, systems-level approaches help identify emergent properties, regulatory bottlenecks, and context-specific vulnerabilities that are not visible from single-gene or single-pathway analyses [224,225]. This perspective is particularly useful for studying nutrition-lncRNA interactions, where small disturbances can propagate through interconnected networks and lead to biologically meaningful results.

### 5.2. Multi-Omics Integration to Map LncRNA-Centered Cancer Regulatory Circuits

Advances in high-throughput technologies have enabled comprehensive profiling of genomic, epigenomic, transcriptomic, proteomic, and metabolomic states in cancer. Integrating these multi-omics datasets is essential for resolving how lncRNAs coordinate responses to nutritional and metabolic cues.

Transcriptomic analyses capture nutrient-induced changes in lncRNA expression, while epigenomic profiling reveals associated alterations in chromatin accessibility, DNA methylation, and histone modifications. Proteomic and interactome datasets identify lncRNA-binding partners and downstream signaling effects, whereas metabolomic profiling links lncRNA regulation to shifts in metabolic flux and redox balance. When analyzed collectively, these datasets enable reconstruction of lncRNA-centered regulatory circuits that connect dietary inputs to cancer-relevant phenotypes [19,226]. Single-cell multi-omics technologies could further refine this framework by resolving cell type-specific lncRNA expression patterns and metabolic states within heterogeneous tumor ecosystems [227]. These approaches could reveal how nutrient-responsive lncRNA networks differ across cancer cells, immune populations, and stromal compartments, providing insights into spatial and functional heterogeneity that influence cancer progression and therapeutic response.

### 5.3. Network Inference and Pathway Modeling of Nutrient–LncRNA Interactions

Computational network inference methods allow systematic identification of regulatory relationships between lncRNAs, signaling pathways, and phenotypic outputs. Correlation-based networks, Bayesian inference models, and causal network reconstruction approaches have been applied to large-scale cancer datasets to identify lncRNAs that function as regulatory hubs or bottlenecks [228,229,230].

In the context of nutrition, these models can incorporate dietary variables, metabolite levels, or nutrient-responsive transcriptional signatures as external inputs. This would enable the identification of lncRNAs that mediate the effects of specific nutrients on oncogenic pathways such as NF-κB, PI3K-AKT-mTOR, Wnt/β-catenin, and TGF-β signaling. Importantly, network modeling will identify how multiple dietary components may converge on shared lncRNA-regulated circuits, reinforcing the concept of coordinated, systems-level modulation rather than isolated molecular effects.

Dynamic modeling approaches could further enable simulation of temporal responses to nutritional perturbations, capturing feedback loops and adaptive responses that influence long-term cancer trajectories. These models would be particularly relevant for chemoprevention, where sustained, low-intensity interventions must be evaluated over extended timescales.

### 5.4. Artificial Intelligence and Machine Learning for Predictive LncRNA-Guided Nutrition Strategies

AI and ML approaches offer powerful tools for extracting predictive insights from complex, high-dimensional datasets [231]. Supervised and unsupervised learning algorithms can be applied to multi-omics and clinical datasets to identify lncRNA signatures associated with dietary exposure, metabolic state, and cancer risk [232]. Feature selection and dimensionality reduction methods can enable prioritization of lncRNAs that are most informative for distinguishing protective versus permissive regulatory states. Graph-based learning models and neural networks can capture non-linear interactions between lncRNAs, metabolites, and signaling pathways, facilitating prediction of context-specific responses to nutritional interventions. AI-driven frameworks can further be leveraged to stratify individuals based on lncRNA expression profiles, metabolic features, and genetic background, supporting precision nutrition strategies aimed at cancer risk modulation. In addition, such models enable in silico testing of hypothetical interventions, accelerating hypothesis generation and reducing reliance on trial-and-error experimental designs [233,234].

However, given the existing body of evidence, AI/ML–based frameworks should be viewed as hypothesis-generating and decision-support tools rather than validated clinical strategies for cancer prevention. While AI and ML approaches can integrate multi-dimensional datasets, including lncRNA expression profiles, metabolic signatures, genetic variation, and nutritional inputs, to identify patterns of nutrient responsiveness, there is currently no direct evidence that AI-guided dietary interventions reduce cancer incidence in humans. Most existing studies are retrospective, preclinical, or focused on short-term molecular or metabolic endpoints rather than long-term cancer outcomes. Importantly, the ability of AI models to predict lncRNA modulation or pathway engagement does not imply causal cancer prevention, and such predictions require rigorous experimental validation. Prospective, longitudinal clinical trials with extended follow-up will be essential to establish whether AI-informed precision nutrition strategies can meaningfully alter cancer risk. Accordingly, we emphasize that current AI-driven models are best suited for prioritizing candidate nutrient–lncRNA interactions, guiding experimental design, and refining biomarker-based stratification strategies, rather than serving as standalone clinical interventions.

### 5.5. Translational Applications: Biomarkers, Liquid Biopsies, and Prevention-Focused Trials

The integration of systems biology and AI approaches position lncRNAs as promising biomarkers for nutrition-informed risk assessment for specific diseases, including cancer [235]. Nutrient-responsive lncRNA signatures can potentially be detected in minimally invasive liquid biopsy platforms, including circulating tumor cells, extracellular vesicles, and cell-free RNA [236]. These signatures may reflect both cancer-associated regulatory states and modifiable metabolic or nutritional exposures.

In translational settings, lncRNA-guided stratification could inform prevention-focused clinical studies by identifying individuals most likely to benefit from targeted dietary interventions. Such approaches may enable rational trial design, improved compliance monitoring, and more precise interpretation of outcomes. Importantly, systems-level models can also identify unintended activation of oncogenic pathways, helping to mitigate risks associated with indiscriminate supplementation.

From a practical standpoint, precision nutrition strategies informed by lncRNA biology are most feasibly implemented through biomarker-guided stratification rather than universal dietary recommendations. Emerging pilot studies and proof-of-concept frameworks suggest that integrating baseline molecular features, such as germline genetic variants, tumor- or tissue-specific lncRNA expression profiles, circulating metabolic markers, and gut microbiome composition, can help identify subgroups of individuals who are more likely to respond to specific nutritional interventions [237,238,239,240]. For example, individuals exhibiting inflammation- or AMPK-associated lncRNA signatures may preferentially benefit from ω-3 fatty acids or polyphenol-rich diets, whereas distinct metabolic or epigenetic profiles may predict responsiveness to methyl-donor–related nutrients [241,242]. In practice, such approaches rely on readily measurable inputs, including blood-based transcriptomic or epigenetic markers, targeted metabolomics, and microbiome-derived metabolites, which can be incorporated into pilot stratification algorithms [237,238,239,240]. Although large-scale clinical validation is still lacking, these early stratified designs demonstrate a feasible path toward matching nutritional interventions to biologically defined responder populations, thereby operationalizing precision nutrition in a manner analogous to biomarker-guided strategies used in targeted cancer prevention and risk modulation.

At present, clinical trials that directly integrate nutritional interventions with lncRNA biomarkers in cancer prevention or interception remain extremely limited. Most human studies linking diet to lncRNA modulation have been conducted outside oncology, primarily in metabolic or inflammatory settings, where circulating lncRNAs have demonstrated responsiveness to defined dietary patterns and utility as predictive or intermediate biomarkers. Recent clinical studies further support this emerging role of lncRNAs in nutrition-associated trials. For instance, in individuals at high risk for type 2 diabetes, circulating lncRNAs such as XIST and LINC01116 were identified as predictive biomarkers of disease incidence, with their epigenetic scores showing positive interactions with adherence to a Mediterranean diet and associated preventive outcomes [243]. In another study, patients with rheumatoid arthritis exhibited elevated levels of the lncRNA NUTM2A-AS1, which correlated with reduced inflammatory markers and suggested dietary modulation of immune regulatory circuits during a 14-day plant-based dietary intervention [244]. While analogous lncRNA-guided nutritional intervention trials in cancer are currently lacking, these non-cancer studies establish the feasibility of incorporating lncRNA-based stratification into human nutritional research. Collectively, these findings underscore the need for future prevention-focused oncology trials that incorporate lncRNAs as molecular readouts or stratification tools rather than as definitive clinical endpoints.

### 5.6. Challenges and Future Directions

Despite significant advances, several challenges must be addressed to fully realize the potential of systems-level lncRNA-guided precision nutrition. These include limited availability of longitudinal, nutrition-annotated multi-omics datasets, variability in lncRNA annotation across platforms, and the need for standardized analytical pipelines. Moreover, causal relationships inferred from computational models require experimental validation in relevant biological contexts. While the translational potential of lncRNA-guided precision nutrition is increasingly supported by mechanistic and pilot human studies, its application to cancer prevention will require rigorous, long-term clinical validation.

Another challenge relates to the contextual role of nutrients as well as their downstream lncRNAs. An important and often underappreciated consideration in nutrition-based modulation of lncRNAs is the pronounced context dependence of their functions and responses. The biological effects of dietary components on lncRNA expression and activity are influenced by multiple variables including cancer type, stage, genetic background, and other individual and environmental factors like age, sex, and nutritional background, which can contribute to diversifying responses to supplementation [245]. For example, folate supplements have a chemopreventive action in normal individuals by altering levels of lncRNA related to one-carbon metabolism and epigenetic stability, but may have deleterious effects in already established cancers by hyper-methylating DNA or altering levels of lncRNA to enhance cell proliferation in such cancers as colorectal cancer or prostate cancer, thus clearly indicating requirements for timing considerations for pre- vs. post-cancer diagnosis [246]. Dose, timing, and duration of exposure further shape these outcomes, underscoring that “more” is not necessarily beneficial. These observations highlight the need for stratified, biomarker-guided nutritional strategies that account for inter-individual variability and disease context, rather than indiscriminate supplementation.

Future efforts should prioritize integrative study designs that combine controlled nutritional interventions with comprehensive lncRNA profiling and systems-level analysis. Such approaches will be essential for translating mechanistic insights into actionable strategies for cancer chemoprevention.

## 6. Therapeutic and Preventive Potential of Nutrition–LncRNA Axis

The convergence of nutritional biology and lncRNA regulation offers tangible opportunities for cancer prevention and early intervention when grounded in specific molecular mechanisms. Rather than serving as broad lifestyle modifiers, dietary bioactive compounds can be viewed as precision inputs capable of reshaping lncRNA-centered regulatory networks that govern oncogenic signaling, metabolic adaptation, inflammation, and cellular plasticity.

### 6.1. Targeting Oncogenic LncRNAs Through Dietary Bioactive Compounds

Several dietary compounds have demonstrated the capacity to repress oncogenic lncRNAs that function as central drivers of cancer progression. For example, polyphenols such as curcumin and resveratrol consistently suppress HOTAIR, a chromatin-associated lncRNA that promotes EMT, metastasis, and epigenetic silencing of tumor suppressor genes. In preclinical cancer models, dietary suppression of HOTAIR is accompanied by reduced invasion, altered chromatin accessibility, and attenuation of EMT-associated transcriptional programs [13,247,248]. These findings suggest that individuals exhibiting elevated HOTAIR expression may represent candidates for lncRNA-guided dietary modulation aimed at limiting early metastatic potential. In this context, resveratrol, by inducing the expression of tumor-suppressive lncRNAs such as GAS5, NBR2, and HOTAIR, may serve as a promising preventive and adjuvant therapeutic agent [112]. Similarly, repression of MALAT1 and NEAT1 by resveratrol, EGCG, and vitamin D intersects with pathways controlling cell cycle progression, stress adaptation, and therapy resistance. Because MALAT1 and NEAT1 are frequently upregulated across multiple tumor types and detectable in circulating RNA pools, their nutrient responsiveness supports both biomarker-driven risk stratification and intervention monitoring in prevention-focused settings [20,81,249,250].

### 6.2. Modulating Inflammatory and Immune-Permissive LncRNA Networks

Chronic inflammation represents a permissive environment for tumor initiation and progression, and several lncRNAs act as key regulators of inflammatory signaling. ω-3 fatty acids provide a clear example of how dietary inputs influence inflammatory lncRNA circuits. Supplementation with EPA and DHA has been associated with suppression of H19 and inflammation-associated lncRNAs that reinforce NF-κB-dependent transcriptional programs [75,111,139,251]. In macrophages and stromal cells, ω-3-responsive lncRNAs modulate polarization states, favoring tumor-suppressive immune contexts over pro-tumorigenic inflammatory signaling. These effects suggest that dietary fatty acid composition may indirectly regulate tumor immune microenvironments through lncRNA-mediated control of cytokine production and immune cell differentiation.

Probiotics and postbiotics have been shown to effectively shift cancer-associated macrophages from the M2 to the anticancer M1 phenotype [252] and to suppress the expression of oncogenic lncRNAs such as PVT1, HOTAIR, MALAT1, and UCA1 (among others) in peripheral blood of rectal cancer patients [209]. Such mechanisms provide a rationale for incorporating lncRNA profiling into studies evaluating dietary modulation of cancer-associated inflammation [253,254,255,256,257].

### 6.3. Metabolic Checkpoint LncRNAs as Leverage Points for Chemoprevention

Metabolic reprogramming is a hallmark of early tumorigenesis, and lncRNAs play critical roles in coordinating energy sensing and anabolic growth. The lncRNA NBR2, which activates AMPK signaling under metabolic stress, functions as a tumor suppressor by restraining mTOR-driven proliferation. Nutrient states that enhance AMPK activity—such as ω-3 fatty acid enrichment or modulation of NAD^+^ metabolism—indirectly reinforce NBR2-dependent growth checkpoints [8,149].

Conversely, oncogenic lncRNAs such as UCA1 promote glycolysis and metabolic flexibility through enhancement of NAD^+^ salvage pathways and PI3K–AKT signaling. Dietary modulation of niacin availability and NAD^+^ flux may therefore influence the balance between tumor-suppressive and tumor-promoting metabolic lncRNA circuits. These examples illustrate how lncRNA-guided metabolic stratification could inform personalized dietary interventions aimed at suppressing early oncogenic metabolic adaptation [34,258,259].

### 6.4. Epigenetic Vulnerability Windows Defined by Folate- and Vitamin D-Responsive LncRNAs

Epigenetic instability represents an early and reversible stage in carcinogenesis, making it particularly amenable to preventive intervention. Folate-dependent one-carbon metabolism influences DNA methylation patterns that regulate lncRNA expression, including oncogenic lncRNAs such as HOTAIR, H19, and UCA1, as well as tumor-suppressive lncRNAs such as MEG3. Balanced folate availability supports methylation-dependent repression of oncogenic lncRNA promoters, whereas deficiency or excess can destabilize these regulatory states [11,134,260,261].

Vitamin D provides an additional example of epigenetic modulation through nuclear receptor–lncRNA networks. VDR-regulated induction of MEG3 and repression of MALAT1 and NEAT1 link vitamin D status to p53 activity, immune regulation, and differentiation programs. These effects suggest that lncRNA-responsive epigenetic windows may exist during which dietary modulation exerts disproportionate influence on cancer risk trajectories [262,263,264].

### 6.5. Toward LncRNA-Guided Precision Prevention Strategies

Together, these examples illustrate how the nutrition–lncRNA axis can be operationalized into mechanistically informed prevention strategies. Rather than universal dietary prescriptions, this framework supports stratifying individuals based on lncRNA expression patterns, metabolic state, and inflammatory tone. Nutritional interventions can then be selected to reinforce tumor-suppressive lncRNA networks or dampen oncogenic circuits.

Importantly, these approaches complement existing prevention and therapeutic strategies. Dietary modulation of lncRNA networks may enhance immune surveillance, reduce inflammatory permissiveness, and sensitize cells to pharmacologic interventions, positioning nutrition as a biological co-modulator rather than an alternative therapy. As multi-omics profiling and systems-level modeling mature, these lncRNA-guided strategies may evolve into actionable tools for reducing cancer risk in defined populations.

## 7. Conclusions

Cancer prevention has historically been constrained by a limited molecular understanding of how modifiable environmental factors interface with the regulatory circuitry that governs tumor initiation and early progression. While epidemiological studies have long implicated diet as a determinant of cancer risk, the absence of a mechanistic framework has hindered translation into actionable, precision-guided strategies. The emergence of lncRNAs as central regulators of transcriptional, epigenetic, metabolic, and immune programs fundamentally reframes this challenge. LncRNAs do not simply accompany oncogenic transformation; they actively integrate genetic context with environmental and metabolic inputs to shape cancer susceptibility.

This review synthesizes growing evidence that nutritional signals—delivered through dietary bioactive compounds, fatty acids, vitamins, probiotics, and micronutrients—consistently converge on lncRNA-centered regulatory networks. Through these networks, nutrition influences core hallmarks of cancer, including EMT, inflammatory permissiveness, metabolic rewiring, stress adaptation, genome stability, and immune surveillance. Importantly, these effects occur at the level of regulatory architecture rather than isolated signaling pathways, providing a mechanistic explanation for how sustained, low-intensity nutritional inputs can exert durable and biologically meaningful effects on cancer-relevant states.

By positioning lncRNAs at the nexus of nutrition and cancer biology, a new paradigm for cancer chemoprevention emerges—one that is mechanistically grounded, context dependent, and inherently aligned with precision medicine. Within this paradigm, nutrition is no longer viewed as a generalized lifestyle modifier but as a programmable biological input capable of reshaping transcriptional and epigenetic landscapes. Nutrient-responsive lncRNAs serve as both effectors and sensors within this system, enabling identification of vulnerable regulatory states that may be intercepted before malignant programs become fixed.

The integration of systems biology, multi-omics profiling, and AI further accelerates the translational potential of this framework. Network-based modeling enables identification of lncRNA regulatory hubs, prediction of pathway-level responses to nutritional modulation, and stratification of individuals according to molecular risk and responsiveness. Importantly, these approaches support prevention-oriented strategies that prioritize early intervention, adaptability, and low toxicity, complementing existing therapeutic modalities rather than competing with them.

Looking forward, translating the nutrition–lncRNA axis into clinical impact will require longitudinal, nutrition-annotated molecular datasets, functional validation in relevant preclinical models, and development of minimally invasive lncRNA-based biomarkers suitable for population-level application. As these efforts mature, lncRNAs may function not only as mechanistic links between diet and cancer biology but also as practical guides for individualized preventive interventions. Importantly, such strategies will require careful context-dependent validation to avoid unintended metabolic or epigenetic consequences.

In summary, the convergence of nutritional science and lncRNA biology provides a coherent and actionable roadmap for precision cancer prevention. Leveraging dietary modulation of lncRNA regulatory networks offers a scalable, biologically rational approach to intercept early oncogenic trajectories and shift the emphasis of cancer control from treatment toward prevention. Embracing this framework has the potential to redefine how nutritional strategies are conceptualized, evaluated, and deployed in the effort to reduce cancer burden. By placing lncRNAs at the intersection of nutrition and cancer biology, this review defines a new paradigm in which lncRNA-guided nutritional modulation becomes a rational and scalable strategy for precision cancer prevention.

## Data Availability

Data supporting the narrative are cited within the paper.

## Abbreviations

The following abbreviations are used in this manuscript:

AI	Artificial intelligence
AMPK	AMP-activated protein kinase
APC	Adenomatous polyposis coli
AKT	Protein kinase B
ATP	Adenosine triphosphate
β-catenin	Beta-catenin
ceRNA	Competing endogenous RNA
DHA	Docosahexaenoic acid
DNMT	DNA methyltransferase
EGCG	Epigallocatechin gallate
EMT	Epithelial–mesenchymal transition
EPA	Eicosapentaenoic acid
FOXO	Forkhead box O
HOTAIR	HOX transcript antisense RNA
IL	Interleukin
lncRNA	Long non-coding RNA
MALAT1	Metastasis-associated lung adenocarcinoma transcript 1
MAPK	Mitogen-activated protein kinase
MEG3	Maternally expressed gene 3
miRNA	MicroRNA
ML	Machine learning
mTOR	Mechanistic target of rapamycin
NAD^+^	Nicotinamide adenine dinucleotide
NAMPT	Nicotinamide phosphoribosyltransferase
NBR2	Neighbor of BRCA1 gene 2
NEAT1	Nuclear paraspeckle assembly transcript 1
NF-κB	Nuclear factor kappa-light-chain-enhancer of activated B cells
PARP	Poly (ADP-ribose) polymerase
PI3K	Phosphoinositide 3-kinase
PPARγ	Peroxisome proliferator-activated receptor gamma
PUFA	Polyunsaturated fatty acid
SAM	S-adenosylmethionine
SIRT	Sirtuin
STAT	Signal transducer and activator of transcription
TGF-β	Transforming growth factor beta
TNF-α	Tumor necrosis factor alpha
UCA1	Urothelial carcinoma-associated 1
VDR	Vitamin D receptor
Wnt	Wingless-related integration site
